# Is It Possible to Predict the Length of Stay of Patients Undergoing Hip-Replacement Surgery?

**DOI:** 10.3390/ijerph19106219

**Published:** 2022-05-20

**Authors:** Teresa Angela Trunfio, Anna Borrelli, Giovanni Improta

**Affiliations:** 1 Department of Advanced Biomedical Sciences, University of Naples “Federico II”, 80131 Naples, Italy; 2 “San Giovanni di Dio e Ruggi d’Aragona” University Hospital, 84121 Salerno, Italy; acquarama@libero.it; 3 Department of Public Health, University of Naples “Federico II”, 80131 Naples, Italy; ing.improta@gmail.com; 4 Interdepartmental Center for Research in Healthcare Management and Innovation in Healthcare (CIRMIS), University of Naples “Federico II”, 80131 Naples, Italy

**Keywords:** data mining, length of stay, hip

## Abstract

The proximal fracture of the femur and hip is the most common reason for hospitalization in orthopedic departments. In Italy, 115,989 hip-replacement surgeries were performed in 2019, showing the economic relevance of studying this type of procedure. This study analyzed the data relating to patients who underwent hip-replacement surgery in the years 2010–2020 at the “San Giovanni di Dio e Ruggi d’Aragona” University Hospital of Salerno. The multiple linear regression (MLR) model and regression and classification algorithms were implemented in order to predict the total length of stay (LOS). Lastly, using a statistical analysis, the impact of COVID-19 was evaluated. The results obtained from the regression analysis showed that the best model was MLR, with an R^2^ value of 0.616, compared with XGBoost, Gradient-Boosted Tree, and Random Forest, with R^2^ values of 0.552, 0.543, and 0.448, respectively. The *t*-test showed that the variables that most influenced the LOS, with the exception of pre-operative LOS, were gender, age, anemia, fracture/dislocation, and urinary disorders. Among the classification algorithms, the best result was obtained with Random Forest, with a sensitivity of the longest LOS of over 89%. In terms of the overall accuracy, Random Forest and Gradient-Boosted Tree achieved a value of 71.76% and an error of 28.24%, followed by Decision Tree, with an accuracy of 71.13% and an error of 28.87%, and, finally, Support Vector Machine, with an accuracy of 65.06% and an error of 34.94%. A significant difference in cardiovascular disease, fracture/dislocation, and post-operative LOS variables was shown by the chi-squared test and Mann–Whitney test in the comparison between 2019 (before COVID-19) and 2020 (in full pandemic emergency conditions).

## 1. Introduction

The proximal fracture of the femur and hip is the most common reason for hospitalization in orthopedic departments. Hip fractures put patients at risk of cardiovascular, pulmonary, thrombotic, infectious, and bleeding complications that can lead to death [1]. The only strategy to prevent immediate negative outcomes is to proceed in a timely manner with surgery. Despite the procedure, however, patients experience increased mortality, health complications, and reduced quality of life [2,3,4].

Although hip fractures account for less than 20% of all osteoporosis-associated fractures [5], considered second only to cardiovascular disease by the World Health Organization [6], they are often used as an indicator of the health of the population and to evaluate the economic impact of this condition. In fact, they account for the majority of morbidity-related and mortality-related health expenditure in men and women over the age of 50 [7,8]. Specifically, globally, 1.3 million fractures were reported in the year 1990, and this figure is estimated to reach 7–21 million by 2050, with an associated expenditure that will reach 9.8 billion USD in the United States and 650 million CAD in Canada [9,10]. These data are associated with the demographic trend, in recent years, of increasing life expectancy, which has changed the age profile of the population. For example, in Italy, the reference country for this study, an increase in life expectancy has been observed in recent years, reaching 79.7 years for men and 84.4 for women [11], with a consequent increase in chronic and degenerative diseases. In the country, about half of the population over 65 has degenerative pathologies of an arthritic nature, with a high impact on motor ability, thus making prosthetic interventions in the orthopedic field among the most frequently performed. In particular, an increase in hip-replacement surgeries (whose elective share amounts to about 2/3) the last five years was recorded, from 104,425 in 2015 to 115,989 in 2019 (+11.1%). In 2020, due to the COVID-19 pandemic, with containment measures such as lockdown and the blocking of elective surgery, there was a marked decrease in the number of cases (N = 96,822), which was quantifiable as 19,167 fewer hospitalizations (−16.5%) compared to the previous year, and a reduction compared to the previous trend figure, which reached 18% (a value corresponding to approximately 21 thousand fewer hospitalizations than expected) [12].

A health care process that involves an increasing number of patients and is transversal, especially when considering patients who are admitted for traumatic fractures [9], must involve effectiveness and efficiency controls to improve not only patient outcomes, but also to ensure the proper use of resources.

A widely used indicator in the literature is the length of stay (LOS). The LOS is an important performance indicator of hospital costs and management. An unnecessary increase in LOS, in addition to affecting resources, exposes patients to nosocomial infections and functional decline [13].

With this in mind, the following work intends to investigate the LOS of patients who underwent hip arthroplasty in the years 2010–2020 at “San Giovanni di Dio e Ruggi d’Aragona” University Hospital of Salerno (Italy). This study was born as an extension of a previous work [14], in which we analyzed a limited number of patients, included in this study, and a limited number of variables. The aim is to build a valid predictive model capable of determining the duration of bed occupancy, based on patients’ clinical and demographic variables, and understanding which are the main factors that influence the total LOS. Finally, the impact of COVID-19 on patients undergoing this procedure is analyzed.

### Related Works

Several studies use advanced data processing in order to support doctors in the prevention, diagnosis, and treatment of diseases [15,16,17,18,19,20,21] or the management of hospital resources [22,23,24,25,26]. In the orthopedic field, many articles study the performance associated with the flow of patients who are admitted for fractures of the lower limbs. For example, Lefaivre et al. determined the effect of delayed surgery on discharge times, in-hospital death, the presence of major and minor medical complications, and the incidence of sores in hip fracture patients. Bracy et al. [27], on the other hand, showed how the institution of orthopedic–hospitalist comanagement (OHC) improves the efficiency of hip-fracture management, as measured by inpatient LOS and time to surgery [28]. Fisher et al. have shown how early mobilization helps reduce the total LOS [29]. With the aim of reducing the total LOS, Fast Tracks were born, a combination of clinical and organizational factors optimized to reduce convalescence and perioperative morbidity, including functional recovery with a consequent reduction in hospitalizations. Husted et al. highlighted the benefits of orthopedic Fast Track in Denmark [30].

Furthermore, in Italy, several studies were conducted to investigate the epidemiology of the problem [31,32] and the choice of prostheses [33], and to improve the process. Scala et al. analyzed how with a Lean Six Sigma approach, a reduction in the total LOS of 39% is achieved for patients admitted with fractures of the femur [34]. Latessa et al. instead used the same methodology to implement Fast Track, with a statistically significant reduction of 12.7% in the LOS [35]. Although there are studies at national and international level that use predictive algorithms for the study of the total LOS [36,37,38,39], there are no other studies in the literature that analyze hip fractures in a large number of patients, including multiple clinical variables and the impact of COVID-19. The hypothesis of this paper is that particular clinical conditions or patient demographics may have a significant impact on LOS and on which healthcare management needs to focus more, to achieve benefits including cost containment considerations. In addition, the COVID-19 pandemic, with all the protocols put in place, may have further affected the process under consideration.

## 2. Materials and Methods

This study analyzed the data relating to patients who underwent hip-replacement surgery in the years 2010–2020 at the “San Giovanni di Dio e Ruggi d’Aragona” University Hospital of Salerno (Italy). Specifically, all patients who had hip surgery as their primary procedure were selected, with the following ICD-9 codes:

- 8151: total hip replacement,
- 8152: partial hip replacement,
- 8153: revision hip replacement

Using the hospital discharge forms, the following information was extracted for the 2515 patients included in the study:

- Age,
- Gender (Male/Female),
- Date of admission, discharge, and principal procedure,
- Main and secondary diagnoses,

Starting from this information, the following independent variables were obtained:

- Gender,
- Age,
- Pre-Operative LOS,
- Diabetes (yes/no),
- Hypertension (yes/no),
- Obesity (yes/no),
- Anemia (yes/no),
- Vitamin D deficiency (yes/no),
- Tumor (yes/no),
- Fracture/Dislocation (yes/no),
- Brain disorders (yes/no),
- Urinary disorders (yes/no),
- Cardiovascular disease (yes/no),
- Respiratory disease (yes/no),
- Anticoagulant therapy (yes/no).

Our data, provided by the Hospital’s Health Department, are completely anonymous, and no personal information is linked or linkable to a specific person. The output is the total LOS in days obtained as the difference between the date of discharge and date of admission. All clinical variables were obtained by analyzing the main and secondary diagnoses reported in the discharge form. Therefore, without a detailed characterization of the clinical picture of each patient, the variables simply indicate the presence (1 Yes) or absence (0 No) of conditions related to that comorbidity. The variable Fracture/Luxation makes it possible to differentiate the proportion of elderly patients who underwent elective surgery from those who suffered a traumatic event.

**Figure 1:**
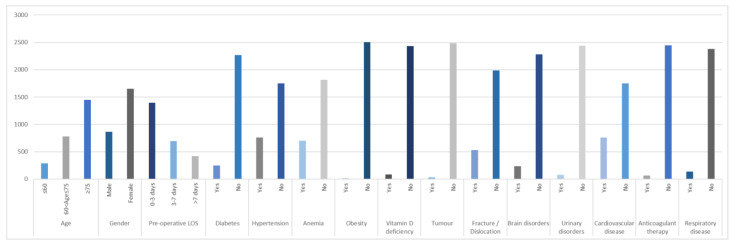
Distribution of the features in the dataset.

Figure 1 shows the distribution of all the variables in the dataset.

### 2.1. Regression and Classification Models

The 15 variables defined above (i.e., gender, age, pre-operative LOS, diabetes, hypertension, obesity, anemia, vitamin D deficiency, tumor, fracture/dislocation, brain disorders, urinary disorders, cardiovascular disease, respiratory disease, and anticoagulant therapy) were used as inputs for the study of total LOS, i.e., the output. The first processing involved the implementation of the MLR model. To this end, IBM SPSS Statistics Version 26.0 software (IBM Corp., Armonk, NY, USA) was used. This software was also used to verify all the preliminary hypotheses on residuals, autocorrelation, the presence of outliers, and the multicollinearity. After this first processing, further regressive algorithms were used, i.e., Random Forest RF, Gradient-Boosted Tree GBT, XGBoost, and Linear Regression LR. RF is a supervised-learning algorithm in which multiple learning algorithms are combined to improve performance. Although it can produce an overfitting, the resulting model is accurate and powerful. GBT is a non-parametric statistical learning algorithm used for both classification and regression problems. As RF, the decision model produced is a set of simple forecasting models, typically decision trees, which are progressively added to each step to improve the result obtained by the previous Weak Learner. The Decision Tree (DT) is a tree-like decision model where the target value is predicted by simple decision rules identified from the data. DTs are simple to understand and require little data preparation, but its disadvantages include overfitting and the creation of biased trees if some classes dominate. XGBoost algorithm is a gradient-boosting algorithm, built through the progressive addition of decision trees in order to improve the performance of the previous tree. In addition, models are fitted using any arbitrary differentiable loss function and gradient descent optimization algorithm. This gives the technique its name, “gradient boosting”, since the loss gradient is minimized as the model is fit, in a similar manner to a neural network. LR is a model that assumes a linear relationship between output and input. Different techniques can be used to prepare or train the linear regression equation from data, the most common of which is called Ordinary Least Squares. Learning, in this case, means estimating the value to be attributed to the coefficients, starting from the available data. Next, the classification algorithms, i.e., Random Forest (RF), Decision Tree (DT), Gradient-Boosted Tree (GBT), and Support Vector Machine (SVM) were implemented. SVM algorithm finds a hyperplane in an N-dimensional space (N—the number of features) that has a maximum margin, i.e., the maximum distance between data points of both classes. To this end, a loss function is used. SVM is effective in high dimensional spaces but it does not directly provide probability estimates. The other algorithms are defined above. This second part was developed with Knime Analytics Platform. For all algorithms, the dataset was broken down into training set and test set, at 80% and 20%, respectively.

### 2.2. Statistical Analysis

To analyze the impact of COVID-19 on the sample under examination, two sub-groups were extracted:

- Group 1: Patients discharged in 2019 and, therefore, before COVID-19.
- Group 2: Patients discharged in 2020 in full pandemic.

Statistical tests were implemented to identify any differences in the two groups. Before proceeding with the selection of the statistical tests, the Kolmogorov–Smirnov test was performed which showed the non-normality of the two distributions. For this reason, the Mann–Whitney U (MW) and chi-squared test with a 95% confidence interval were used.

## 3. Results

**Figure 2:**
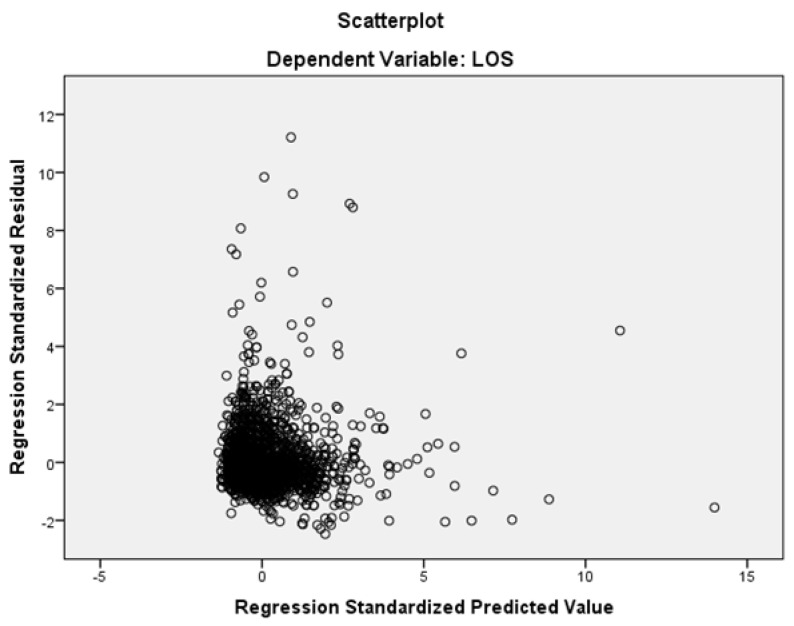
Homoscedasticity of the data.

Preliminary to the elaboration, the hypotheses underlying the implementation of the MLR model were verified. The Durbin–Watson test had an output of 1.934. The test always has a value ranging between 0 and 4. A value of 2.0 indicated that there was no autocorrelation detected in the sample. Continuing with the analysis of the residuals, from the graph showing “standardized expected value regression” on the x-axis against “standardized residual regression”, shown in Figure 2, a random distribution around zero was observed, which supported the hypothesis of homoscedasticity. The residuals therefore had a constant variance.

**Figure 3:**
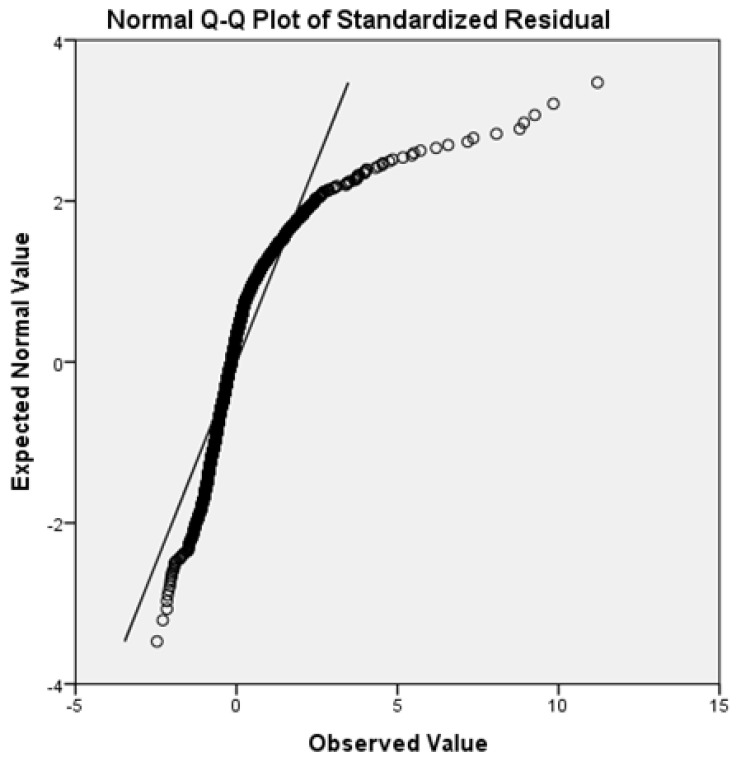
Q–Q plot.

Concluding the residual analysis, the Quartile–Quartile plot (Q–Q plot) presented in Figure 3 was used to evaluate the distribution trend. If the two sets came from a population with the same distribution, the points were expected to fall approximately along this reference line. The greater the departure from this reference line, the greater the evidence for the conclusion that the two data sets came from populations with different distributions.

Although the curve did not exactly retrace the ideal line, the slight variation did not affect the good performance of the model.

Before implementing the model, the absence of multicollinearity was tested using the Pearson correlation and the tolerance and variance inflation factor (VIF), while the presence of outliers was determined through the calculation of Cook’s distance. Table 1 shows the results of the Pearson correlation.

The results of the Pearson correlation showed that the LOS had the highest correlation with the pre-operative LOS, included by definition in LOS, while for the other variables, the correlation was always lower than 0.7.

For the tolerance and VIF, the former always assumed a value greater than 0.2, while the latter was always less than 10, suggesting the absence of multicollinearity. Lastly, Cook’s distance was always less than 1.

Having verified the hypotheses, the MLR model was implemented.

Table 2 shows an R^2^ value just above the 0.5 threshold, showing that it was quite representative of the specific case study. Table 3 shows the details of the coefficients and the *t*-test applied to the variables with a significance of 95%.

The results of the *t*-test highlighted that gender, age, pre-operative LOS, anemia, fracture/dislocation, and urinary disorders were significantly correlated with the total LOS. Standardized coefficients help to compare the effect of each individual independent variable to the dependent variable. In this case, assuming the value 0 when comorbidities were absent, a patient with anemia conditioned the dependent variable more by having the highest beta coefficient associated with it, if the pre-operative LOS was excluded. In addition, according to the beta column, women (gender: 1 male/2 female) with advanced age, as this was a continuous variable, significantly influenced the dependent variable of the model.

In addition to the MLR model, further regression algorithms were tested. Table 4 shows the results obtained in terms of R^2^ and root mean squared error.

**Figure 4:**
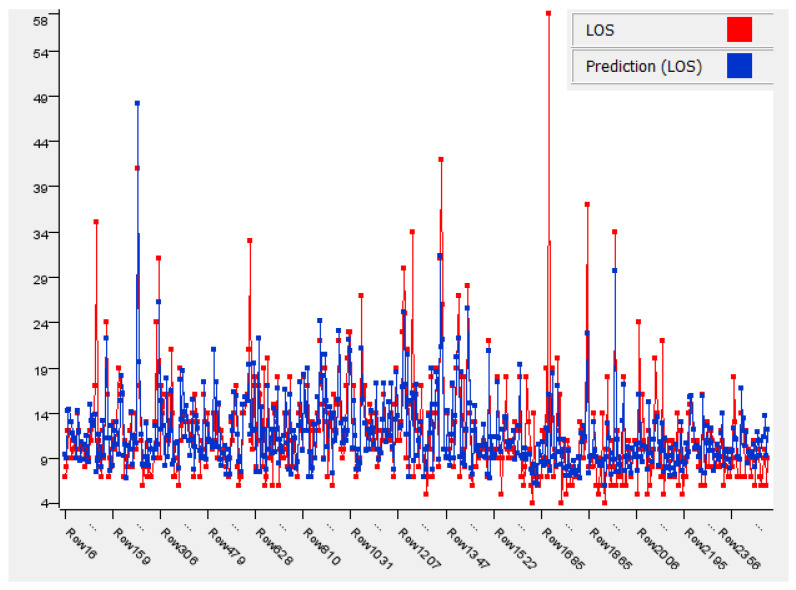
Linear Regression.

**Figure 5:**
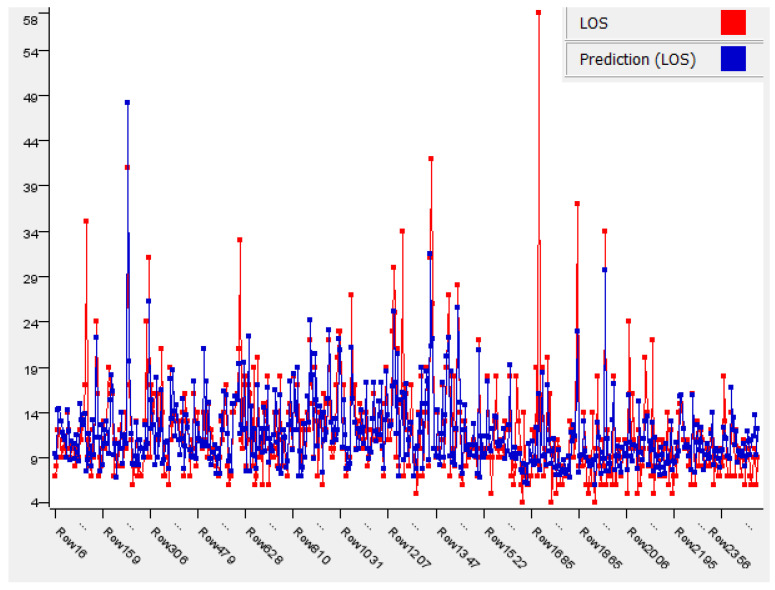
XGBoost.

Among the algorithms, XGBoost and LR had the best performance, with an R^2^ value of 0.552, followed by GBT, with 0.543, and, finally, RF, with 0.448. However, even the best value of R^2^, obtained with XGBoost/LR, did not improve the performance of the MLR model. The results obtained with the best algorithms used are shown in graphic form in Figure 4 and Figure 5.

After the regression models, four different classification algorithms were tested. For implementation, the LOS was divided into three categories, as indicated below:

1. LOS ≤ 6 days.
2. 6 days < LOS ≤ 12 days.
3. LOS > 12 days.

Table 5 shows the results obtained.

With an accuracy of 71.76% and an error of 28.24%, RF and GBT had the best performance, followed by DT, with an accuracy of 71.13% and an error of 28.87%, and, finally, SVM, with an accuracy of 65.06% and an error of 34.94%. For all the algorithms, optimal results were not achieved in all three categories. The results, however, showed a high ability to predict longer LOS, which weigh heavily on healthcare costs. The details of the classification for the best algorithm are shown in Table 6.

**Figure 6:**
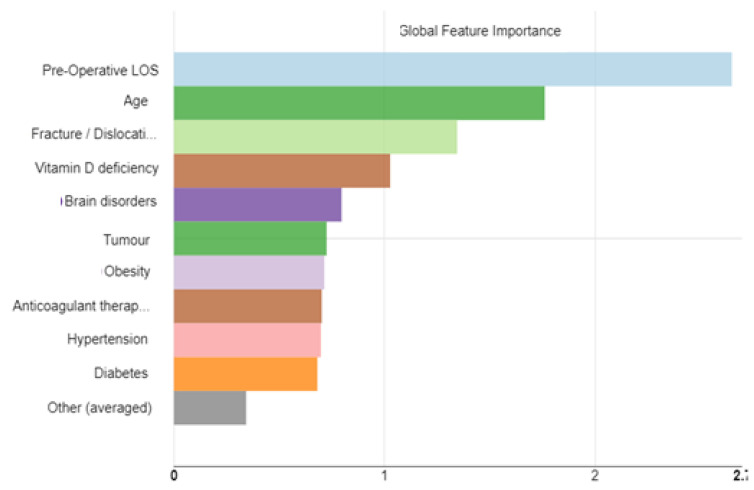
Global importance Feature.

To analyze the global feature importance, a Global Surrogate Random Forest was used. Global Surrogate Random Forest is a Random Forest model trained to approximate the predictions of already implemented RF models. Random Forest is trained on standard pre-processed input data with optimized parameters “tree depth”, “number of models,” and “minimum child node size”. The surrogate model was trained successfully. Specifically, focusing on class 3, that is, the one to which the longest stay corresponded, which was the one that was of greatest relevance to health management, the model returned an accuracy of 0.942, and the overall significance characteristic shown in Figure 6.

Among the variables that most affected the model from class 3, in accordance with the specific procedure analyzed, excluding the pre-operative LOS, were age, fracture/dislocation and vitamin D deficiency. Gender, anemia, and urinary disorders, which in the MLR model were significantly related to total hospitalization, in this case, had a non-significant impact and were included in the variable, other.

Lastly, the impact of COVID-19 on the model parameters was analyzed. Specifically, the pre-COVID-19 (year 2019) and during-COVID-19 (year 2020) data were compared using statistical analysis. The results are reported in Table 7.

The statistical tests highlighted a significant difference in cardiovascular disease, fracture/dislocation, and post-operative LOS.

## 4. Discussion

In this study, a set of variables was analyzed in order to be able to predict the LOS for hip-replacement surgery. The analysis was conducted at “San Giovanni di Dio e Ruggi d’Aragona” University Hospital of Salerno (Italy), analyzing the data recorded from 2010 to 2020.

### 4.1. Results of Regression and Classification Models

This work is an extension of a previous work, published by the same research group, in which MLR and ML algorithms were used to investigate the LOS only for the years 2019–2020 [14]. Using this previous article as a reference, the same tools were used in this study. The results obtained for the regression models showed that the best was MLR, with an R^2^ value of 0.616, which was slightly lower than the previous result, of 0.687. The model was therefore quite representative of the case study in which it was implemented. The statistical test instead showed that the variables that most influence the model, with the exception of the pre-operative LOS, which by, definition depends on it, were gender, age, anemia, fracture/dislocation, and urinary disorders. This result was in line with those previously reported in the literature. For example, Ricci et al. [40] and Latessa et al. [35] highlighted a different LOS according to gender, while Scala et al. [34] showed an influence of cardiovascular diseases. Husted et al. [41], on the other hand, showed that age, sex, comorbidity, and pre- and post-operative hemoglobin levels influence post-operative outcomes in general, including LOS and patient satisfaction, while Calgue et al. [42] showed that significant effects are also due to the type of fracture.

The classification models did not show significant results for the three categories envisaged by the work. With an accuracy of 71.76% and an error of 28.24%, RF and GBT had the best performance, which did not reach the accuracy of over 83% obtained by GBT in [14]. Although the model as a whole could not be validated, the confusion matrix showed the high capacity of the model in predicting cases with LOS greater than 14 days. This is strategically important for healthcare facilities, as these are the cases that have the greatest impact on resource consumption and healthcare costs.

### 4.2. COVID-19’s Impact

The impact of the SARS-CoV-2 pandemic on the sample was analyzed. Comparing the same variables for the year 2019 (pre-COVID-19) and the year 2020 (during COVID-19), the statistical tests highlighted a significant difference in terms of cardiovascular disease, fracture/dislocation, and post-operative LOS. In particular, there was an increase in the number of patients undergoing surgery with cardiovascular comorbidities or a diagnosis of fracture/dislocation. This, unlike the results reported in the literature [35,41,42], did not cause an increase in postoperative LOS, which actually decreased. This phenomenon can be explained both by the protocols put in place to contain the pandemic and limit the time spent in hospital and by the reduced number of beds, which were mostly dedicated to COVID patients.

### 4.3. Uniqueness of the Present Study, Clinical Implications, and Limitations

The strength of the work is that it considers a large number of data and variables that help to further characterize the sample, also including the changes caused by the pandemic. The ability to understand which variables have the greatest impact on the LOS can help healthcare managers to allocate resources or implement specific pathways, such as fast tracks [30], for privileged access to treatment and the elimination of inefficiencies.

However, this work is not without limitations. In particular, the effect that multiple procedures have on LOS is not considered, and the results cannot be generalized, since this is a single-center study. In addition, variables that could be used to analyze the socioeconomic status of the patients were not included, and the data source, hospital discharge records, did not allow the precise characterization of the degree of severity of the comorbidities studied.

## 5. Conclusions

In this study, the data of 2515 patients undergoing hip-replacement surgery at “San Giovanni di Dio e Ruggi d’Aragona” University Hospital of Salerno (Italy) in the years 2010–2020 were processed using regression and classification models. Both elaborations showed that the variables that most influenced the LOS were age and the presence of fracture/dislocation. These results, together with the good performance of the models, could be used by healthcare managers to create specific pathways, according to the age or the main diagnoses that lead to interventions. This can help both bed management, through LOS prediction and turnover planning, but also all other hospital resources. The analysis of the impact of COVID-19, therefore, could be an important pointer to capture the inadvertent positive effects of the pandemic from an organizational perspective, such as the establishment of specific protocols that led to the effective and efficient use of hospital facilities.

Future developments will include the implementation of additional data processing and classification techniques, focusing in more detail on patients’ pathways and how they have changed due to the pandemic. Furthermore, additional variables will be included in the models in addition to the specific characterization of those already provided.

## Figures and Tables

**Table 1:** Pearson correlation.

		LOS	Gender	Age	Pre-Operative LOS	Diabetes	Hypertension	Obesity	Anemia	Vitamin D Deficiency	Tumor	Fracture/Dislocation	Brain Disorders	Urinary Disorders	Cardiovascular Disease	Respiratory Disease	Anticoagulant Therapy
Pearson Correlation	LOS	1.000	0.054	0.137	0.772	−0.027	−0.104	−0.023	0.049	−0.054	0.069	0.248	−0.009	0.046	0.109	0.024	0.002
	Gender	0.054	1.000	0.182	−0.010	−0.008	0.080	0.029	0.104	0.040	−0.008	0.055	0.011	−0.035	−0.016	−0.085	−0.029
	Age	0.137	0.182	1.000	.088	0.060	0.189	−0.018	0.126	0.115	−0.005	0.095	0.119	0.077	0.218	0.054	0.064
	Pre-operative LOS	0.772	−0.010	0.088	1.000	−0.064	−0.161	−0.022	−0.064	−0.101	0.072	0.260	−0.019	0.005	0.078	−0.002	−0.008
	Diabetes	−0.027	−0.008	0.060	−0.064	1.000	0.202	−0.020	0.090	0.036	0.052	−0.024	0.028	0.033	0.066	0.079	0.068
	Hypertension	−0.104	0.080	0.189	−0.161	0.202	1.000	0.062	0.174	0.130	−0.039	−0.142	0.058	0.062	0.177	0.112	0.066
	Obesity	−0.023	0.029	−0.018	−0.022	−0.020	0.062	1.000	0.007	−0.011	−0.006	−0.031	−0.019	0.028	0.004	−0.014	0.031
	Anemia	0.049	0.104	0.126	−0.064	0.090	0.174	0.007	1.000	0.154	0.033	−0.029	0.090	0.089	0.063	0.055	0.066
	Vitamin D deficiency	−0.054	0.040	0.115	−0.101	0.036	0.130	−0.011	0.154	1.000	0.001	−0.052	0.125	0.005	0.072	0.083	0.024
	Tumor	0.069	−0.008	−0.005	0.072	0.052	−0.039	−0.006	0.033	0.001	1.000	0.017	0.004	0.024	0.042	0.105	−0.018
	Fracture/Dislocation	0.248	0.055	0.095	0.260	−0.024	−0.142	−0.031	−0.029	−0.052	0.017	1.000	−0.041	−0.019	0.202	−0.042	−0.050
	Brain disorders	−0.009	0.011	0.119	−0.019	0.028	0.058	−0.019	0.090	0.125	0.004	−0.041	1.000	−0.018	0.040	0.038	0.014
	Urinary disorders	0.046	−0.035	0.077	0.005	0.0033	0.062	0.028	0.089	0.005	0.024	−0.019	−0.018	1.000	0.067	0.008	0.027
	Cardiovascular disease	0.109	−0.016	0.218	0.078	0.066	0.177	0.004	0.063	0.072	0.042	0.202	0.040	0.067	1.000	0.040	0.183
	Respiratory disease	0.024	−0.085	0.054	−0.002	0.079	0.112	−0.014	0.055	0.083	0.105	−0.042	0.038	0.008	0.040	1.000	0.025
	Anticoagulant therapy	0.002	−0.029	0.064	−0.008	0.068	0.066	0.031	0.066	0.024	−0.018	−0.050	0.014	0.027	0.183	0.025	1.000
Sig. (1-tailed)	LOS		0.003	0.000	0.000	0.089	0.000	0.120	0.007	0.003	0.000	0.000	0.326	0.011	0.000	0.110	0.465
	Gender	0.003		0.000	0.308	0.341	0.000	0.071	0.000	0.023	0.340	0.003	0.284	0.040	0.218	0.000	0.071
	Age	0.000	0.000		0.000	0.001	0.000	0.190	0.000	0.000	0.402	0.000	0.000	0.000	0.000	0.004	0.001
	Pre-operative LOS	0.000	0.308	0.000		0.001	0.000	0.132	0.001	0.000	0.000	0.000	0.177	0.394	0.000	0.451	0.352
	Diabetes	0.089	0.341	0.001	0.001		0.000	0.160	0.000	0.036	0.005	0.117	0.082	0.048	0.000	0.000	0.000
	Hypertension	0.000	0.000	0.000	0.000	0.000		0.001	0.000	0.000	0.026	0.000	0.002	0.001	0.000	0.000	0.000
	Obesity	0.120	0.071	0.190	0.132	0.160	0.001		0.354	0.289	0.373	0.060	0.169	0.083	0.421	0.235	0.060
	Anemia	0.007	0.000	0.000	0.001	0.000	0.000	0.354		0.000	0.050	0.076	0.000	0.000	0.001	0.003	0.000
	Vitamin D deficiency	0.003	0.023	0.000	0.000	0.036	0.000	0.289	0.000		0.482	0.005	0.000	0.392	0.000	0.000	0.114
	Tumor	0.000	0.340	0.402	0.000	0.005	0.026	0.373	0.050	0.482		0.194	0.420	0.118	0.017	0.000	0.183
	Fracture/dislocation	0.000	0.003	0.000	0.000	0.117	0.000	0.060	0.076	0.005	0.194		0.021	0.166	0.000	0.017	0.006
	Brain disorders	0.326	0.284	0.000	0.177	0.082	0.002	0.169	0.000	0.000	0.420	0.021		0.189	0.022	0.028	0.236
	Urinary disorders	0.011	0.040	0.000	0.394	0.048	0.001	0.083	0.000	0.392	0.118	0.166	0.189		0.000	0.352	0.090
	Cardiovascular disease	0.000	0.218	0.000	0.000	0.000	0.000	0.421	0.001	0.000	0.017	0.000	0.022	0.000		0.022	0.000
	Respiratory disease	0.110	0.000	0.004	0.451	0.000	0.000	0.235	0.003	0.000	0.000	0.017	0.028	0.352	0.022		0.107
	Anticoagulant therapy	0.465	0.071	0.001	0.352	0.000	0.000	0.060	0.000	0.114	0.183	0.006	0.236	0.090	0.000	0.107	

**Table 2:** Multiple linear regression model.

	R	R^2^	Adjusted R^2^	Std. Error of the Estimate
Model	0.785	0.616	0.613	3.726

**Table 3:** Coefficients of MLR model.

	Unstandardized Coefficients		Standardized Coefficients	t	*p*-Value
	B	Std. Error	Beta		
(Constant)	4.405	0.522	-	8.442	0.000
Gender	0.609	0.162	0.048	3.762	**0.000**
Age	0.020	0.007	0.040	2.960	**0.003**
Pre-operative LOS	1.011	0.017	0.760	57.908	**0.000**
Diabetes	0.221	0.257	0.011	0.862	0.389
Hypertension	−0.166	0.178	−0.013	−0.933	0.351
Obesity	−0.624	1.250	−0.006	−0.499	0.618
Anemia	1.130	0.173	0.084	6.537	**0.000**
Vitamin D deficiency	0.127	0.430	0.004	0.295	0.768
Tumor	0.328	0.705	0.006	0.465	0.642
Fracture/dislocation	0.593	0.196	0.040	3.020	**0.003**
Brain disorders	−0.159	0.261	−0.008	−0.610	0.542
Urinary disorders	1.115	0.433	0.032	2.572	**0.010**
Cardiovascular disease	0.348	0.176	0.027	1.983	**0.048**
Respiratory disease	0.632	0.335	0.024	1.888	0.059
Anticoagulant therapy	−0.116	0.470	−0.003	−0.248	0.804

**Table 4:** Results of regression algorithms.

	LR	RF	GBT	XGBoost
R^2^	0.552	0.448	0.543	0.552
Root mean squared error	3.843	4.497	3.883	3.843

**Table 5:** Performance metrics of all selected algorithms.

Performance Metrics	Class	DT	GBT	RF	SVM
Accuracy (%)	Overall	71.13	71.76	71.76	65.06
Error (%)	Overall	28.87	28.24	28.24	34.94
Precision (%)	1	65.35	69.49	55.04	63.46
	2	61.58	60.93	80.68	61.29
	3	89.19	89.66	75.14	67.69
Sensitivity (%)	1	64.34	63.57	76.34	76.74
	2	71.02	74.43	59.17	32.39
	3	76.30	75.14	89.66	89.60
Specificity (%)	1	87.39	89.68	84.94	83.67
	2	74.17	72.19	85.71	88.08
	3	94.75	95.08	87.09	75.74
F-measure (%)	1	64.84	66.40	63.96	69.47
	2	65.96	67.01	68.27	42.38
	3	82.24	81.76	81.76	77.11

**Table 6:** Random Forest confusion matrix.

Real/Predicted	1	2	3
1	71	20	2
2	57	142	41
3	1	14	130

**Table 7:** Analysis of COVID-19 impact.

Variable	2019 N = 272	2020 N = 185	*p*-Value
Age			
Mean	77.76	78.22	0.800
Gender			
Male	88	59	0.918
Female	184	126	
Pre-operative LOS			
Mean	3.05	3.14	0.066
Post-operative LOS			
Mean	7.70	7.09	**0.040**
Diabetes			
No	233	155	0.582
Yes	39	30	
Hypertension			
No	159	101	0.413
Yes	113	84	
Anemia			
No	168	117	0.749
Yes	104	68	
Obesity			
No	268	185	0.098
Yes	4	0	
Vitamin D deficiency			
No	225	154	0.884
Yes	47	31	
Tumor			
No	264	180	0.880
Yes	8	5	
Fracture/dislocation			
No	262	142	**0.000**
Yes	10	43	
Brain disorders			
No	218	155	0.325
Yes	54	30	
Urinary disorders			
No	261	177	0.883
Yes	11	8	
Cardiovascular disease			
No	192	101	**0.000**
Yes	80	84	
Anticoagulant therapy			
No	257	178	0.396
Yes	15	7	
Respiratory disease			
No	243	174	0.080
Yes	29	11	
LOS			
Mean	10.75	10.22	0.240

## Data Availability

The datasets generated and/or analyzed during the current study are not publicly available for privacy reasons, but they are available from the corresponding author on reasonable request.
